# Supplementary material for: Anillin propels myosin-independent constriction of actin rings
Source: Nat Commun. 2021 Jul 28;12:4595. doi: 10.1038/s41467-021-24474-1 (PMC8319318; doi:10.1038/s41467-021-24474-1)
Supplement: Supplementary file 1 — Supplementary Information [file 41467_2021_24474_MOESM1_ESM.pdf]

Supplemental Information

Anillin propels myosin-independent constriction of actin rings

Ondřej Kučera[1,†], Valerie Siahaan[1], Daniel Janda[1], Sietske H. Dijkstra[1], Eliška Pilátová[1], Eva Zatecka[1], Stefan Diez[2,3,4], Marcus Braun[1*], Zdenek Lansky[1*]

[1] Institute of Biotechnology, Czech Academy of Sciences, BIOCEV, Vestec, Prague West, Czechia.

[2] B CUBE – Center for Molecular Bioengineering, TU Dresden, Dresden, Germany.

[3] Max Planck Institute of Molecular Cell Biology and Genetics, Dresden, Germany.

[4] Cluster of Excellence Physics of Life, Technische Universität Dresden, Dresden, Germany

†Present address: CytoMorpho Lab, Laboratoire Physiologie Cellulaire & Végétale, Institut de recherche interdisciplinaire de Grenoble, Commissariat à l'énergie atomique et aux énergies alternatives (CEA), Grenoble, France.

* marcus.braun@ibt.cas.cz, zdenek.lansky@ibt.cas.cz

Contents :

Figs. S1 – S5

Supplementary Tables 1 and 2

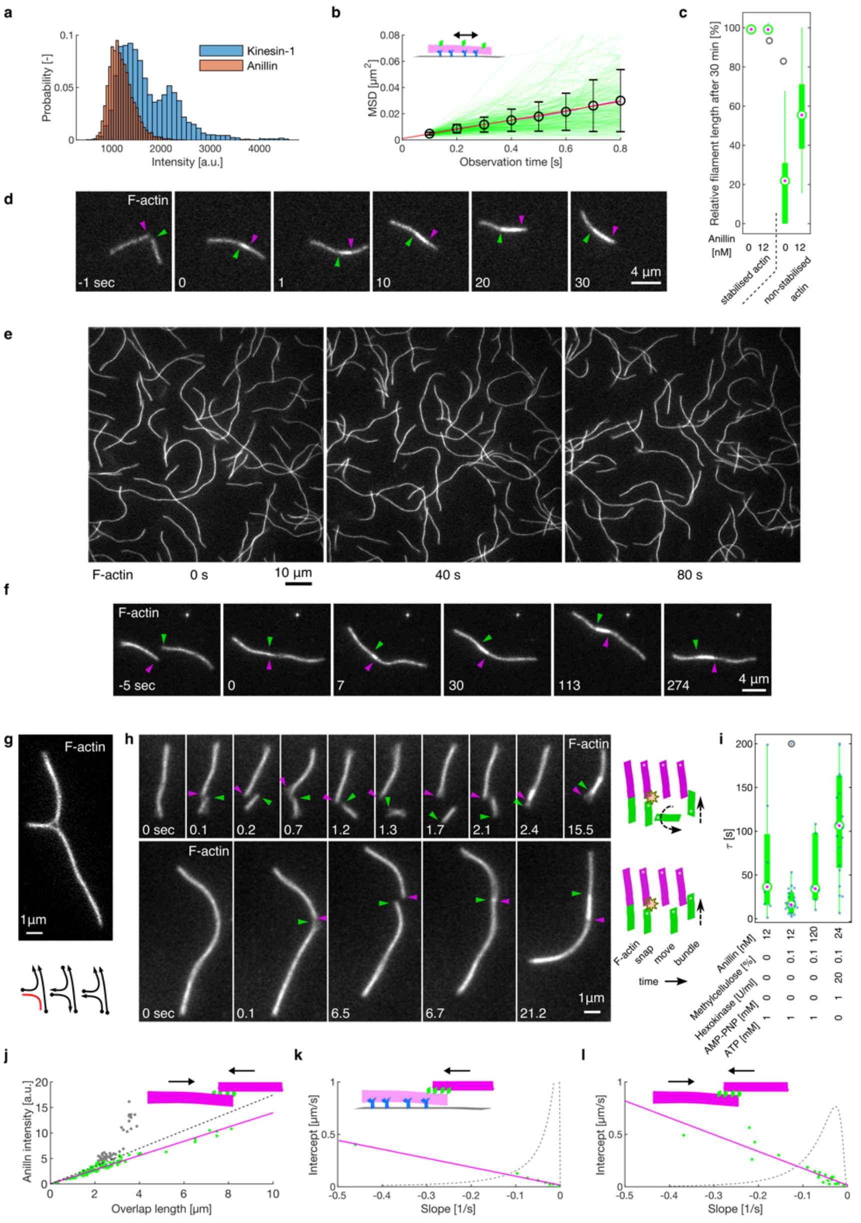

Fig. S1.

a, Histograms of the fluorescence intensity of single dimeric GFP-labelled kinesin-1 molecules bound to a microtubule (blue) and anillin-GFP particles bound to an actin filament (red). The histogram of the homodimeric kinesin-1-GFP, comprising two GFP molecules, shows typical bimodal shape, while that of anillin-GFP is mono-modal suggesting that anillin is in monomeric state (128 anillin-GFP molecules in 2 replicates). **b**, Mean-squared displacement (MSD) of anillin-GFP molecules diffusing on actin filaments. Green curves show MSD traces of individual molecules (n = 268 molecules in 2 experiments), the error bars show mean ± SD. Red line represents a linear fit to the mean of the MSD. **c**, Relative change of the lengths of actin filaments in the absence and presence of anillin-GFP (n = 13, 17, 25, and 24 filaments in 2 experiments). Filament lengths are shown as the percentage of their initial length, indicating that the presence of anillin does not influence the length of phalloidin-stabilized filaments and slows down the disassembly of non-stabilized actin filaments. **d**, Time-lapse fluorescence micrographs showing directional sliding of actin filaments in the presence of 120 nM anillin-

GFP. **e**, Actin filaments do not bundle in the absence of anillin (see Movie 4). Time-lapse micrograph shows that actin filaments are free to move in the lateral plane, but do not form bundles when anillin is absent, showing that the concentration of methylcellulose used in experiments with no immobilised filaments is low enough to not induce actin bundling through depletion effects [34]. **f,** Time-lapse fluorescence micrographs showing directional sliding of actin filaments in the presence of 24 nM anillin-GFP, 20 units/ml hexokinase, and 1 μM AMP-PNP (in the absence of ATP). **g**, **h**, Anillin can bundle and slide both parallel and antiparallel actin filaments. **g**, A construct of three filaments bundled by anillin-GFP in which every filament overlaps partially with each other. Below is a schematic illustration showing that both, parallel and antiparallel filament pairs are formed, in agreement with previously published data [31]. **h**, Time-lapse fluorescence micrographs showing initially a single actin filament in the presence of anillin (not visualized). After the filament broke due to thermal fluctuations, the two parts connected again in an antiparallel (top) and parallel (bottom**)** manner, respectively, and started sliding along each other. The arrowheads indicate the newly formed filament ends formed by the breaking of the initial filament. A schematic representation of the events is shown to the right. **i**, Comparison of the time constant (representing the sliding velocity) of the directional sliding events at the conditions used in this study (n = 8, 24, 6, and 20 events in at least 2 experiments). Corresponding data points overlay the boxplots. **j**, Intensity of anillin-GFP in the overlap during relative sliding of unbound actin filaments. The amount of anillin-GFP in the overlap between two filaments scales linearly with the overlap length. The dots represent individual timepoints (n = 187 time points in 16 events, 5 experiments). The data were fitted by a linear trend (dashed line). Two events with unusually higher intensity were marked as outliers (grey dots). The linear trend of remaining events (green dots, n = 142 time points in 14 events, 4 experiments) is shown in solid line (magenta). **k**, **l**, Dependence between the parameters of the linear fit to the data, such as shown in Fig. 1 k (sliding velocity as a function of the overlap length) in experiments using immobilised actin filaments (**k**, n = 8 bundles in 5 experiments) and mobile actin filaments in the presence of methylcellulose (**l**, n = 24 bundles in 15 experiments). Although the events had random initial overlap length, the contraction in all events followed a regular pattern with linear dependence between the extrapolated maximum sliding velocity (intercept) and the deceleration rate (slope). Green dots show data from individual sliding events, solid magenta line is a linear trend, and the dotted curve shows an estimate of the exponential distribution of the slope of the fit. In **c** and **i**, data are represented as boxplots. Central marks represent median, top and bottom edges of the box indicate the 75th and 25th percentiles, respectively. Whiskers extend the most extreme points that are not considered outliers. Outliers are marked as grey circles.

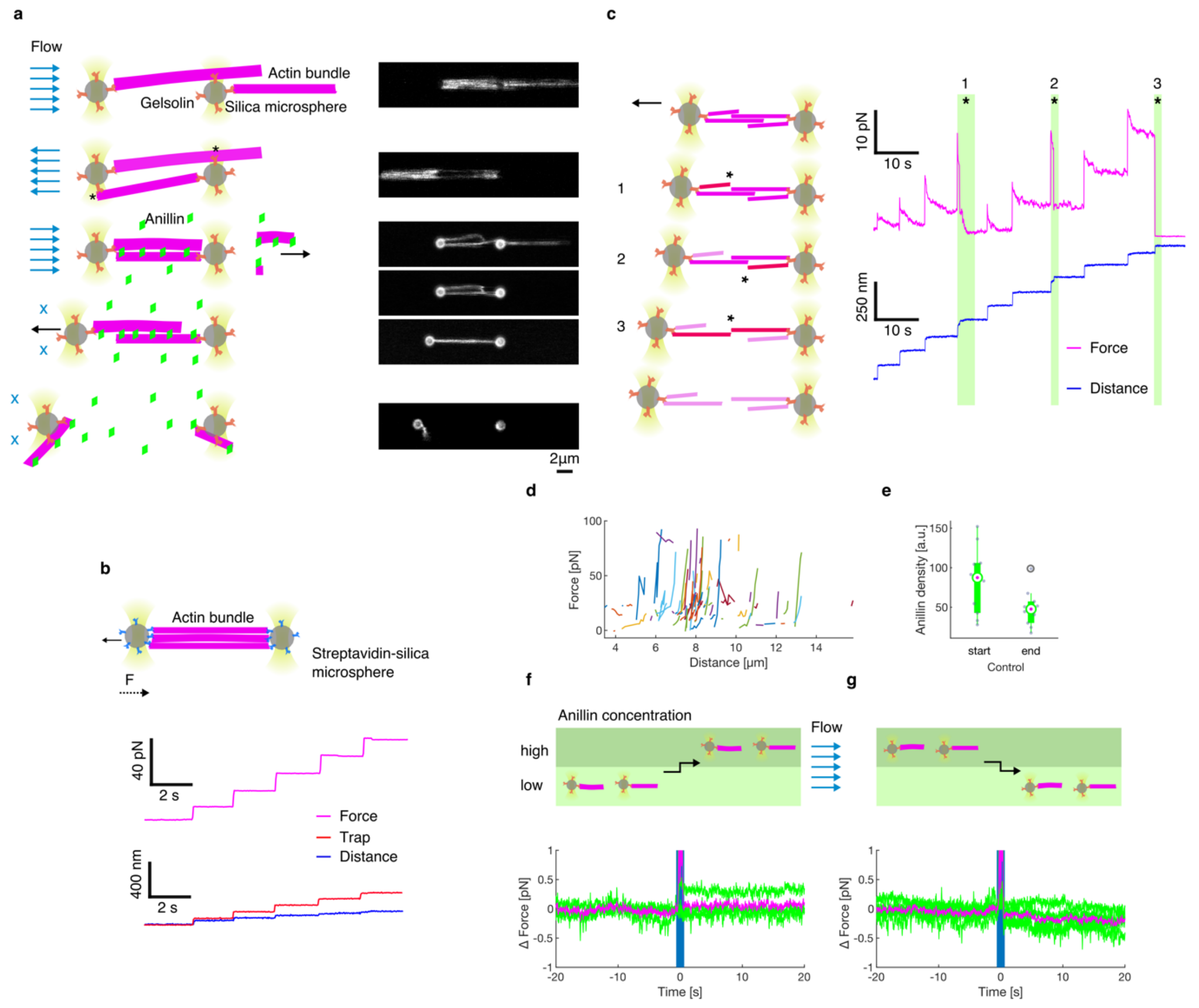

Fig. S2.

a, Schematics and fluorescence micrographs showing how the anillin-actin bundle was created in the optical tweezers; similarly as done in [35]. Briefly, the barbed ends of the filaments firstly bind gelsolin-coated microspheres. Then, by reversing the buffer flow, the filaments are transiently attached (indicated by asterisk) to the opposite microsphere. The construct is then moved to anillin-GFP channel where the higher concentration of $CaCl_2$ triggers severing activity of gelsolin. As anillin binds the filaments at the same time, its bundling activity prevents the construct from breaking up (Methods)**. b**, Control experiment showing the force time trace (top) and the distance between the beads (middle) in response to step-wise movement of the left microsphere (bottom) when the actin bundle was bridging both microspheres (biotin-phalloidin stabilised actin filaments suspended between two streptavidin microspheres). In contrast to Fig. 2 d, the force time trace increases in steps concomitant with the microsphere position steps. The steps in detected force are not followed by relaxation, showing that the actin filaments in the bundle form a solid bridge between the two microspheres and cannot slide relative to each other. **c**, Temporal profile of the force response of an actin-anillin bundle to the stepwise increase of the distance between the beads. The sudden drops of the force, which we interpret as an actin filament sliding apart completely from the rest of the bundle, are highlighted in green and numbered. **d**, Steady state asymptotical force values of all measured force-distance curves of anillin-bundled actin filaments. Individual experiments are colour-coded (n = 43 actin-anillin bundles in 43 experiments). **e**, Fluorescence intensity of anillin-GFP

near the beads (the non-connected part of the bundle) was used for the estimate of photobleaching in the experiment (n = 10 experiments). Corresponding data points overlay the boxplots. **f**, **g**, Control experiments showing the force time trace when the actin-decorated but disjoined microspheres were moved between two channels with different concentration of anillin-GFP. Moving the disjoined construct between microfluidic channels with a stationary laminar flow (schematic depiction on the top) does not cause changes in the force response (bottom) - compare to Fig. 2 f, g. Green curves represent individual measurements (n = 8 events in 3 experiments) with mean shown in magenta. In **f**, data are represented as boxplots. Central marks represent median, top and bottom edges of the box indicate the 75th and 25th percentiles, respectively. Whiskers extend the most extreme points that are not considered outliers. Outliers are marked as grey circles.

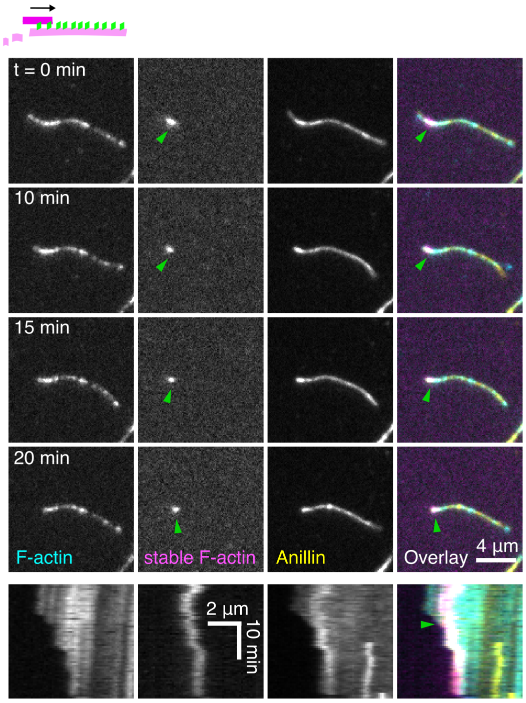

Fig. S3.

Time-lapse fluorescence micrographs showing a short, stabilized filament (indicated by a green arrowhead) crosslinked by anillin-GFP to a long non-stabilized actin filament undergoing disassembly. Initially the stabilized filament diffuses along the non-stabilized filament. When the end of the disassembling actin filament reaches the stabilized filament, the stabilized filament follows the retreating tip of the non-stabilized actin filament. Corresponding kymographs are shown below. The moment when the end of the disassembling actin filament reaches the stabilized filament is indicated by a green arrowhead in the kymograph (see Movie 5).

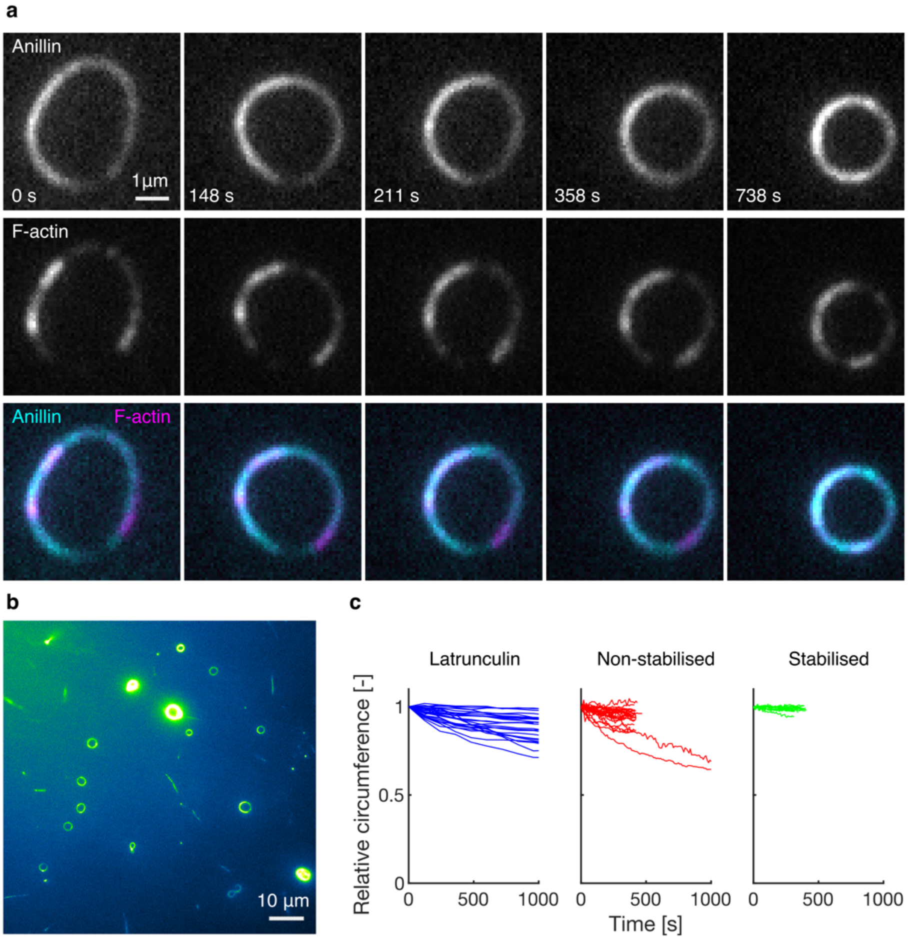

Fig. S4.

a, Time-lapse micrograph of the constriction of the anillin-actin ring composed of non-stabilised actin filaments, showing the anillin-GFP and rhodamine-actin channels and their overlay (see Movie 8). The experiment was repeated 16 times with similar results. **b**, Fluorescence micrograph showing anillin-actin structures formed within the microfluidic channel. Only rings were used for further analysis. This observation was typical for all conditions used (33 experiments). **c**, Temporal profiles of the relative circumference of the anillin-actin rings observed in this study.

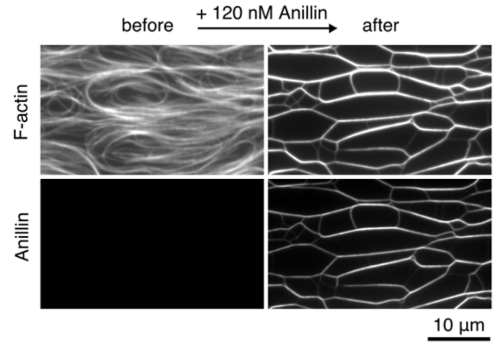

Fig. S5.

Fluorescence micrograph of the formation of an actin-anillin network upon addition of 120 nM anillin to a dense solution of actin filaments. This experiment was repeated five-times with similar results.

Supplementary Table 1

**Primer name**	**Target**	**PCR**	**Sequence** (bold: complementary to gene, italic: restriction site)	**Conc. [µM]**	**use**	**PCR template vector**	**Destination Vector**	**recipient backbone**	**restriction sites generated**
**oDL167**	hsAnillin	fwd	aataataacat*GCGGCCGC*A**ATGgatccgtttacggagaaactgc**	100	PCR	pDL148	pDL150	pOCC8	Asc1 / Not1
**oDL168**	hsAnillin	rev	aataataacat*GGCGCGCC****aggctttccaataggtttgtagcaagc***	100	PCR	pDL148	pDL150	pOCC8	Asc1 / Not1

Supplementary Table 2

**Protein ID**	**Intensity**	**% in sample**
Anillin-C-EGFP-HIS6	18229000000	61.55659872
Myosin-II (A0A2H1WPK0)	44521000	0.150340739

Please refer to the supplementary data set for the full version of the table.
