# Supplementary material for: Anillin propels myosin-independent constriction of actin rings
Source: Nat Commun. 2021 Jul 28;12:4595. doi: 10.1038/s41467-021-24474-1 (PMC8319318; doi:10.1038/s41467-021-24474-1)
Supplement: Supplementary file 11 — Description of Additional Supplementary Files [file 41467_2021_24474_MOESM11_ESM.docx]

Description of additional supplementary files

Title: Movie 1.

Description: Diffusive motion of mobile actin filaments crosslinked by anillin (not visualized) to long, sparsely fluorescently labelled, immobilised actin filaments.

Title: Movie 2.

Description: Anillin-driven sliding of a mobile actin filament (bright) along an immobilised filament (dim).

Title: Movie 3.

Description: Anillin-driven sliding of two mobile actin filaments.

Title: Movie 4.

Description: Actin filaments do not bundle in the absence of anillin.

Title: Movie 5-7.

Description: Anillin couples with actin disassembly to generate directed filament sliding.

Title: Movie 8.

Description: Constriction of the anillin-actin ring composed of non-stabilised actin filaments. Overlay of the anillin-GFP (cyan) and rhodamine- actin (magenta) fluorescence channels.
