# Supplementary material for: Anillin propels myosin-independent constriction of actin rings
Source: Nat Commun. 2021 Jul 28;12:4595. doi: 10.1038/s41467-021-24474-1 (PMC8319318; doi:10.1038/s41467-021-24474-1)
Supplement: Supplementary file 12 — Reporting Summary [file 41467_2021_24474_MOESM12_ESM.pdf]

nature research

Corresponding author(s): Zdenek Lansky
Marcus Braun

Last updated by author(s): June 2, 2021

Reporting Summary

Nature Research wishes to improve the reproducibility of the work that we publish. This form provides structure for consistency and transparency in reporting. For further information on Nature Research policies, see our Editorial Policies and the Editorial Policy Checklist.

Statistics

For all statistical analyses, confirm that the following items are present in the figure legend, table legend, main text, or Methods section.

n/a	Confirmed	
☐	☒	The exact sample size (*n*) for each experimental group/condition, given as a discrete number and unit of measurement
☐	☒	A statement on whether measurements were taken from distinct samples or whether the same sample was measured repeatedly
☐	☒	The statistical test(s) used AND whether they are one- or two-sided<br>*Only common tests should be described solely by name; describe more complex techniques in the Methods section.*
☒	☐	A description of all covariates tested
☒	☐	A description of any assumptions or corrections, such as tests of normality and adjustment for multiple comparisons
☐	☒	A full description of the statistical parameters including central tendency (e.g. means) or other basic estimates (e.g. regression coefficient) AND variation (e.g. standard deviation) or associated estimates of uncertainty (e.g. confidence intervals)
☐	☒	For null hypothesis testing, the test statistic (e.g. *F*, *t*, *r*) with confidence intervals, effect sizes, degrees of freedom and *P* value noted<br>*Give P values as exact values whenever suitable.*
☒	☐	For Bayesian analysis, information on the choice of priors and Markov chain Monte Carlo settings
☒	☐	For hierarchical and complex designs, identification of the appropriate level for tests and full reporting of outcomes
☒	☐	Estimates of effect sizes (e.g. Cohen's *d*, Pearson's *r*), indicating how they were calculated

Our web collection on statistics for biologists contains articles on many of the points above.

Software and code

Policy information about availability of computer code

Data collection	Data were collected using NikonNIS Elements Ar v5.02, Scanary v. 3.3.0, LUMICKS, The Netherlands, TrapStepper v. 3.6.1, LUMICKS, The Netherlands, BlueLake v 1.5, LUMICKS, The Netherlands
Data analysis	Data were analyzed using FIJI 2.0.0-rc-67/1.52c, Matlab R2018a 9.4.0.813654, FIESTA 1.6.0, MaxQuant (version 1.6.10.43), Perseus 1.6.10.43

For manuscripts utilizing custom algorithms or software that are central to the research but not yet described in published literature, software must be made available to editors and reviewers. We strongly encourage code deposition in a community repository (e.g. GitHub). See the Nature Research guidelines for submitting code & software for further information.

Data

Policy information about availability of data

All manuscripts must include a data availability statement. This statement should provide the following information, where applicable:

- Accession codes, unique identifiers, or web links for publicly available datasets
- A list of figures that have associated raw data
- A description of any restrictions on data availability

The dataset, including the source data underlying the figures, is deposited at figshare, https://doi.org/10.6084/m9.figshare.14725188. Further information and requests for reagents can be directed to and will be fulfilled by Zdenek Lansky (zdenek.lansky@ibt.cas.cz).

Field-specific reporting

Please select the one below that is the best fit for your research. If you are not sure, read the appropriate sections before making your selection.

☒ Life sciences ☐ Behavioural & social sciences ☐ Ecological, evolutionary & environmental sciences

For a reference copy of the document with all sections, see nature.com/documents/nr-reporting-summary-flat.pdf

Life sciences study design

All studies must disclose on these points even when the disclosure is negative.

Sample size	Sample sizes were chosen within the consensus in the field; meaning that during each independent experiment (i.e. in each individual flow channel), microscopy fields of view were randomly chosen. In each field of view, the number of molecules, filament bundles or other filament structures present, determined the sample size per measurement. If experimentally possible, several fields of view per flow channel were imaged, increasing the sample size and randomizing the experiment. Sample sizes are limited by experimental feasibility.
Data exclusions	Na data were excluded.
Replication	Experiments were performed over several months by several experimentalists, all replication attempts (that were not impeded by unrelated events, like image acquisition software malfunction) were successful. For each quantified experiment and each exemplary image or kymograph 'n' describes the number of biologically individual samples (individual molecules, filaments, etc.). Details are given in the figure legends.
Randomization	Biological samples (proteins and filaments) as well as all other assay components were taken randomly in small volumes by pipetting from stock solutions of larger volumes and allocated randomly to the different experimental groups. During experiments, random fields of view were chosen, for analysis either all events in a field of view or all events on a random filament were chosen.
Blinding	Investigators were necessarily blinded to group allocation as it is impossible to influence or select any sub-population of biological samples (proteins, actin filaments or any other assay components) that are taken from the stock solutions during pipetting.

Reporting for specific materials, systems and methods

We require information from authors about some types of materials, experimental systems and methods used in many studies. Here, indicate whether each material, system or method listed is relevant to your study. If you are not sure if a list item applies to your research, read the appropriate section before selecting a response.

Materials & experimental systems

n/a	Involved in the study
☐	☒ Antibodies
☐	☒ Eukaryotic cell lines
☒	☐ Palaeontology and archaeology
☒	☐ Animals and other organisms
☒	☐ Human research participants
☒	☐ Clinical data
☒	☐ Dual use research of concern

Methods

n/a	Involved in the study
☒	☐ ChIP-seq
☒	☐ Flow cytometry
☒	☐ MRI-based neuroimaging

Antibodies

Antibodies used	anti-biotin antibody - B3640, Sigma (diluted to 1mg/ml in PBS for use)
Validation	The antibody was solely used to immobilize the actin filaments to the coverslip surface. The actin filament sample was purified, meaning that only actin was present in the sample. The fact that the filaments bound to the antibody-coated surface thus validates the use of this antibody for this application. Importantly, the antiboty was not used for identification of proteins.

Eukaryotic cell lines

Policy information about cell lines

Cell line source(s)	Cell lines were exclusively used for recombinant protein production.<br><br>Commercial Sf9-ESF insect cells were purchased from Expression systems, Davis, CA, USA (#94-001F, ATCC CRL-1711)
Authentication	Commercial cells were not authenticated.

Mycoplasma contamination	Sf9 cell cultures were routinely tested for Mycoplasma contamination using the Mycoplasma Detection Kit from ATCC (# 90-1001K). All tests were negative.
Commonly misidentified lines<br>(See ICLAC register)	No commonly misidentified cell lines were used in this study.
